# Erratum: Corrections to Two Articles in *European Journal of Pediatric Surgery Reports* , Volume 13, Issue 1

**DOI:** 10.1055/a-2706-9632

**Published:** 2025-09-26

In *European Journal of Pediatric Surgery Reports* , Volume 13, Number 1, the following articles were published with incorrect page numbers (DOI:  and  ). The page numbers have been corrected subsequently in both articles with the publication of this erratum. The publisher regrets this error.:

- Zheng Y, Wu R, Chen R, Xiong S, Yun J, Peng W. Kaposiform Hemangioendothelioma with Kasabach–Merritt Phenomenon in a Neonate: A Case Report. Eur J Pediatr Surg Rep 2025;13(01):e51–e54
- Massaguer C, Jorge IDH, García LS, Ortells JP, Fernández MEM, Tarrado X. Vanishing Gastroschisis: The Importance of Prenatal Diagnosis in a Seemingly Normal Abdomen. Eur J Pediatr Surg Rep 2025;13(01):e55–e58

